# Hand Foot Syndrome Has the Strongest Impact on QOL in Skin Toxicities of Chemotherapy

**DOI:** 10.7150/jca.31059

**Published:** 2019-08-27

**Authors:** Ryuta Urakawa, Masahito Tarutani, Kazumi Kubota, Etsuko Uejima

**Affiliations:** 1 Department of Pharmacy, Osaka University Dental Hospital, 1-8 Yamada-oka, Suita, Osaka, Japan; 2 Graduate School and School of Pharmaceutical Sciences, Osaka University, 1-6 Yamada-oka, Suita, Osaka, Japan; 3 Department of Dermatology, Kinki Central Hospital, 3-1 Kurumazuka, Itami, Hyogo, Japan; 4 Department of Biostatistics, Yokohama City University School of Medicine, 3-9 Fukuura, Kanazawa-ku, Yokohama, Kanagawa, Japan

**Keywords:** quality of life, skin toxicity, chemotherapy

## Abstract

**Background**: Chemotherapy often results in dermatologic toxicities, which decrease quality of life (QOL) of cancer patients. These adverse skin reactions sometimes happen simultaneously. Though previous reports have demonstrated that skin reactions influence QOL, those reports were focused on only one kind of skin toxicity or on the most serious skin toxicity. The aim of this study is to demonstrate the contribution of each skin toxicity to QOL.

**Methods**: This is a cross-sectional study at Kinki Central Hospital. Patients were enrolled who underwent skin toxic chemotherapy from April 1 to June 30, 2017. DLQI and Skindex29 were used to grade the QOL of patients. Also, the severity of skin toxicities was evaluated based on National Cancer Institute Common Terminology Criteria for Adverse Events version 4.0 (NCI-CTCAE ver4.0). We investigated how QOL changed with patient demographic and clinical characteristics, the worst skin toxicity grade, and each skin toxicity using statistical analyses.

**Results**: No significant differences were detected between QOL scores (total score of DLQI, emotions domain, symptoms domain, functioning domain and total score of Skindex29) and patient demographic and clinical characteristics (P values were 0.155, 0.086, 0.052, 0.312 and 0.114, respectively). There were statistically significant QOL differences among the grades of the worst skin toxicity (P values were <0.001). Xerosis, paronycia, pigmentation, and hand foot syndrome showed statistically significant associations with some QOL domains analyzed by multiple logistic regression analyses adjusted by demographic characteristics. When adjusted by both demographic characteristics and other skin toxicities, three of xerosis, paronycia, and pigmentation showed no statistically significant associations, but hand foot syndrome showed statistically significant associations in all subdomains and total score of Skindex29 (P values were <0.05).

**Conclusions**: Hand foot syndrome was a stronger factor in decreasing QOL than xerosis, paronychia, pigmentation, or rash. Therefore, especially in hand foot syndrome, prevention, early detection, and daily medical care are necessary to maintain QOL.

## Introduction

Chemotherapeutic agents can cause diverse skin toxicities. Hand foot syndrome is often caused by multikinase inhibitors and capecitabine 1, 2, 3. Rash and paronychia are commonly observed adverse effects of epidermal growth factor receptor (EGFR) tyrosine kinase inhibitors (TKIs) 4, 5. Xerosis and pigmentation are often seen under the influence of many of the skin toxic chemotherapeutic agents 5, 6. These adverse skin reactions usually occur simultaneously and result in decreased quality of life (QOL) in cancer patients under chemotherapy.

Dermatology Life Quality Index (DLQI) and Skindex29 are dermatology-specific measurement tools often used in assessment of psoriasis patients 7, 8, 9. Some reports have adopted DLQI or Skindex to evaluate QOL of patients treated with skin-toxic chemotherapeutic agents 10-17. Those reports have examined negative impact on QOL by chemotherapy, or positive impact by elaborate monitoring plus prophylactic or reactive management of skin toxicities. Because past reports were focused on only one kind of skin toxicity or the most serious of skin toxicities, QOL score could be established from an unexpected skin toxicity which had the strongest impact on QOL 10-16, 18, 19. So it is necessary to find out which skin toxicity influences QOL most strongly. Here we established the hypothesis that skin disorders caused by anticancer drugs have different effects on patient QOL depending on their type. By using DLQI and Skindex29, we verified which skin disorder affected QOL and to what extent.

## Materials and Methods

### Patient Selection

The cancer patients were enrolled who underwent skin toxic chemotherapy at Kinki Central Hospital from April 1 to June 30, 2017. The eligibility criteria included minimum age 18 years, an Eastern Cooperative Oncology Group performance status of 0 or 1, and adequate competency to answer the QOL questions appropriately. Skin toxic chemotherapeutic agents consisted of capecitabine, TS-1, cetuximab, panitumumab, gefitinib, erlotinib, afatinib, osimertinib, regorafenib, everolimus, and axitinib. Cancer type and duration from initial administration were not included in the criteria. Exclusion criteria were the patients with skin diseases at the beginning of the research due to factors other than side effects of anticancer drugs. Institutional review board approved this study protocol, the informed consent form, and QOL assessment materials. Before being enrolled in this study, patients signed the informed consent form after getting sufficient explanations.

### Study Objectives

The objective in this study was to investigate which skin toxicities influenced QOL and to what extent. Associations between mean QOL scores and mean grades of skin toxicities were evaluated.

### Study Design

This is a cross-sectional study at Kinki Central Hospital.

### Patient Demographic and Clinical characteristics

Patients were classified by age, gender, and their chemotherapy regimen. Patients were divided into two groups by median age. Chemotherapy regimens were classified into five groups: capecitabine-based regimens, EGFR-TKI, regimens not containing capecitabine but containing cetuximab or panitumumab, regimens containing capecitabine and panitumumab, and others.

### Grading and QOL assessment of skin toxicities

Adverse effects were assessed using the assessment sheet based on National Cancer Institute Common Terminology Criteria for Adverse Events version 4.0 (NCI-CTCAE ver4.0). QOL scores were calculated using DLQI and Skindex29. Patients completed the adverse effect assessment sheet and question sheets of DLQI and Skindex29 on a consultation day. Pharmacists or nurses confirmed the severity of adverse effects based on the assessment sheet and medical examination by interviewing the patients. Finally, medical doctors who were blinded to patients' participation in this study confirmed the severity of adverse effects. Skin toxicities were classified into rash, xerosis, paronychia, pigmentation, and hand foot syndrome. The worst grade of all classified skin toxicities was categorized as the worst skin toxicity.

### Statistical Analysis

Means comparisons between patient demographic and clinical characteristics and QOL scores were examined using nonparametric statistical method. Mean comparisons between age or gender and QOL scores were examined using unpaired two tailed t-test. Mean comparisons between regimens and QOL scores were examined using Kruskal-Wallis test. Also, mean comparisons between the worst skin toxicity grades and QOL scores were examined by Kruskal-Wallis test. Mann-Whitney Bonferroni correction was performed for comparisons among significantly different QOL domains after Kruskal-Wallis test. Associations between QOL scores and grades of each skin toxicity were examined using multiple logistic regression analyses adjusting for demographic and clinical characteristics, and for those characteristics and adverse effects together. The significant level of all statistical test results was evaluated at two-sided alpha level of 0.05. Statistical analyses were performed based on SPSS ver.24.

## Results

A total of 67 patients were enrolled in this study, and all of them completed the toxicities assessment sheet and QOL evaluation sheets. Patient demographic and clinical characteristics are summarized in Table 1. The mean age of patients was 71.0 years. Of the 67 patients, 37 (55.2%) were male and 30 (44.8%) were female. Chemotherapy regimens patients received were as follows: 32 capecitabine based without EGFR antibody regimens, 18 EGFR-TKI regimens, 6 cetuximab or panitumumab plus other chemotherapeutic agent regimens, 3 capecitabine based plus panitumumab regimens, and 8 other regimens.

Number of patients with each grade of skin toxicity is shown in Table 2. Eighteen patients had no skin toxicities, and 49 patients had some skin toxicities. One patient had grade3 xerosis, 3 patients had grade3 paronychia, 4 patients had grade3 hand foot syndrome, and total 5 patients had some grade3 skin toxicities.

### QOL Scores by Patient Demographic and Clinical Characteristics

There were no statistically significant differences between QOL scores and age, gender. No statistically significant differences were observed between QOL scores and chemotherapy regimens (Table 3).

### Comparisons between QOL Scores and Worst Skin Toxicity Grades

All the QOL domains showed statistically significant differences among the grades of worst skin toxicities (Table 4). Mann-Whitney Bonferroni correction showed statistically significant differences with the QOL domains and worst skin toxicity grades. All domains showed statistically significant differences between grade0 and grade2 or grade3. Total score of DLQI, emotions domain, functioning domain, and total score of Skindex29 showed statistically significant differences between grade1 and grade2.

### Associations between QOL Scores and Worst Skin Toxicities

Associations between QOL Scores and Skin Toxicities are shown in Table 5. Xerosis, paronycia, pigmentation, and hand foot syndrome showed statistically significant associations with some QOL domains analyzed by multiple logistic regression adjusted by demographic characteristics. When adjusted by both demographic characteristics and other skin toxicities, three of xerosis, paronycia, and pigmentation showed no statistically significant associations, but hand foot syndrome showed statistically significant associations in all subdomains and total score of Skindex29. The odds ratios of QOL decrement by hand foot syndrome were as follows: emotions domain (odds ratio 2.509, 95% confidence interval 1.127-5.586, p =0.024), symptoms domain (odds ratio 4.511, 95% confidence interval 1.721-11.825, p =0.002), functioning domain (odds ratio 2.545, 95% confidence interval 1.115-5.812, p =0.027), and total score of Skindex29 (odds ratio 5.203, 95% confidence interval 1.698-15.943, p =0.004). Rash showed no statistically significant associations with any QOL domains. DLQI showed statistically significant associations with xerosis, paronycia, pigmentation, and hand foot syndrome in the demographic adjusted models, but no statistically significant associations were observed in the fully adjusted models.

## Discussion

Chemotherapy sometimes causes side effects of skin disorders such as hand foot syndrome, rash, paronychia, xerosis and pigmentation 1-6. Not only do those skin disorders make it difficult to continue treatment, they can also cause impairment of the patient's quality of life 10-19. Therefore, when using anticancer drugs with skin toxicity, prevention, early detection and treatment of skin disorders are essential 11, 13, 20, 21.

The purpose of this study was to investigate which skin disorders have a large impact on QOL in daily medical practice where many skin disorders coexist. As a result, it was found that hand foot syndrome had a greater influence on QOL than rash, xerosis, paronychia, or pigmentation. In addition, our study suggests that Skindex29 is a tool with higher detection power than DLQI as a skin specific QOL measurement tool.

There are many previous reports about relationship between skin disorders and quality of life. There have been some reports that focused on the worst grade skin disorder and decreased quality of life 10-14, 18, 19. Some reports have found negative impact on QOL 10-12 and the others have not 13, 14, 18, 19. In our study, negative impact was obtained. From the results of the Mann-Whitney test after Kruskal-Wallis test, there were statistically significant differences among Grades 0 and 2 or 3 and between Grades 1 and 2. Therefore, it is suggested that there is a large QOL gap between Grade 1 and Grade 2, and that it is significant to prevent skin disorder not less than Grade 2 in the clinical setting. However, it is not yet known which skin dysfunction affected QOL reduction and must be avoid worsening especially.

We analyzed the influence of each side effect on QOL by logistic regression analysis. As a result, when only the patient background was corrected, xerosis, paronychia, pigmentation, and hand foot syndrome had statistically significant differences, affecting quality of life, but when corrected to include each side effect, the statistically significant effect on QOL was found only in hand foot syndrome. Hence, it was shown that when hand foot syndrome develops, QOL decreases more than with other skin disorders. Therefore, in the case of drugs that are likely to cause hand foot syndrome, prevention, early detection, and early treatment of hand foot syndrome are considered essential for maintaining patient QOL. There is a study which has shown that gender might play an important role on QOL in the perception of skin disorders 12. Another study has shown that younger patients have had lower overall dermatology-related QOL than older patients 15. But from our result, there were no relationships between QOL and gender or age. In these cases, it is possible that there are gender or age differences in their works or lifestyles between countries and regions. There is a report that the patients with employment status and more highly education have been the statistically significant factors lead to reduction of QOL after multivariable analyses 10. Because these factors were not verified in our study, we could not include them in analyses. But these reports suggest the necessity to correct the patient background in order to analyze the relationship between skin disorder and QOL. Also, another previous research has reported the results of association between many kinds of skin toxicities and QOL 17. Though it has concluded that rash and pruritus produced the greatest negative impact, it was failed to adjust for selected confounding variables. Our study might to be said to reveal for the first time that hand foot syndrome influences the true outcome greatly when we discuss the relationship between QOL and skin toxicities.

Nevertheless, significant differences could not be obtained as a result of logistic regression analysis with all factors corrected in the DLQI used this time. However, Skindex29 was able to detect significant difference in each subdomain. It was therefore suggested that Skindex29 is a QOL measurement tool with higher detection power than DLQI. These results suggest the possibility that Skindex29 has better sensitivity to clinical severity than DLQI, as previously described 7. However, DLQI is more convenient than Skindex29 in terms of the number of questions, and it seems that it is necessary to choose according to the intended use rather than which one is superior.

There are four limitations in our study. The first is the method for assessing the severity of adverse events. Scores of DLQI have been statistically significantly high when self-reported acne severity is severe, whereas those scores have been not statistically significantly different when assessed by medical doctor, as previously reported 8. This result might reflect the different viewpoints on acne severity between patients and medical staffs. Additionally, severity assessment using NCI-CTCAE during chemotherapy has been different among patients, nurses, and clinicians as previously described 22. In our study, severity was assessed in three steps, the first was reported by patients, the second was assessments by pharmacists or nurses, and the last was confirmation by medical doctors who were blinded to patients' participation in this study. Because severity change might have changed the result of this study, this study was performed in accordance with practical steps to get a realistic result.

Nationality was another limitation in this study because of differences in lifestyle and cultural differences in describing quality of life. Because this study was conducted at one institution in Japan, it might result in a different outcome in other countries. Yet our findings offer some contributions in measuring QOL during chemotherapy.

The third is sample size. Because this study was conducted with only 67 patients in single hospital in Japan, the number of cases was possibly insufficient. It is desirable to increase the possibility of generalization by calculating an appropriate sample size before implementation and targeting patients in a wide area. In the future, it is necessary to increase the number of patients studied by using multicenter cooperative clinical research methods.

The fourth is validation of study findings. This study was conducted on any cancer patients with any skin toxic chemotherapy because we focused on the relationships between QOL and kinds of skin reaction. Since we did not excluded patients other than those who originally had skin diseases, this result is applicable to many patients treated with skin toxic anticancer drugs. Also, because severities of skin reaction are confirmed by blinded medical doctors and QOL scores were reported by patients, it is considered that subjectivity does not affect the research results. But as described above, limitations of nationality and sample size can affect the validity. Additionally, the use of DLQI and Skindex29 has another possible factor of weaken the validity because uniform standard tool for assessment of dermatology specific QOL does not exist. But some useful tools including DLQI and Skindex have been used and have recommended in many previous studies 10-17.

In conclusion, our hypothesis that skin disorders caused by anticancer drugs have different effects on patient QOL depending on their type proved correct. Our study revealed that hand foot syndrome was a stronger factor in decreasing QOL than xerosis, paronychia, pigmentation, or rash. This result means that especially in hand foot syndrome, prevention, early detection, and daily medical care are required. This study also shows the testing power of Skindex29 in measuring QOL for chemotherapy-related skin toxicities. But because of convenience of DLQI, DLQI or Skindex29 should be selected depending on the purpose and the situation of research.

## Figures and Tables

**Table 1:** Patient Demographic and Clinical Characteristics

Characteristics	N=67
Age	
Mean (SD)	71.0 (8.4)
Gender	
Male n (%)	37 (55.2%)
Female n (%)	30 (44.8%)
Regimen	
Capecitabine based	32
EGFR-TKIs (gefitinib, elrotinib, afatinib, osimertinib)	18
Cetuximab, Panitumumab+chemotherapy agents	6
Capecitabine + Panitumumab	3
Others	8

**Table 2:** Number of Patients with Each Grade of Skin Toxicity (NCI-CTCAE ver4.0)

Skin toxicity	Grade0	Grade1	Grade2	Grade3
Rash	53	10	4	0
Xerosis	41	19	6	1
Paronychia	46	13	5	3
Pigmentation	54	13	-	-
Hand foot syndrome	42	11	10	4
Worst skin toxicity	18	30	14	5

**Table 3:** QOL Scores by Patient Demographic and Clinical Characteristics

QOL domain	Age		Gender		Regimens					P
	<71	71≤	Male	Female	X based	EGFR-TKI	Cmab, Pmab	X+Pmab	Others	
	(n=35)	(n=32)	(n=37)	(n=30)	(n=32)	(n=18)	(n=6)	(n=3)	(n=8)	
Total (DLQI)	2.49 (2.97)	2.97 (3.86)	2.03 (2.60)	3.57 (4.08)	1.78 (2.20)	3.33 (3.33)	4.33 (3.56)	4.67 (4.51)	3.13 (6.10)	0.155
Emotions	12.71 (15.49)	16.25 (17.45)	11.49 (13.21)	18.00 (19.31)	8.44 (11.53)	22.08 (18.91)	16.67 (16.41)	25.00 (22.22)	15.31 (19.61)	0.086
Symptoms	18.78 (16.89)	25.45 (19.41)	20.17 (16.86)	24.17 (20.02)	16.29 (13.79)	28.37 (18.68)	33.93 (15.44)	36.90 (37.51)	15.63 (19.56)	0.052
Functioning	7.92 (12.76)	10.09 (12.35)	6.70 (9.98)	11.74 (14.79)	6.05 (11.17)	12.62 (13.99)	10.07 (12.60)	13.89 (14.63)	9.64 (13.82)	0.312
Total (Skindex29)	12.19 (13.97)	15.92 (15.01)	11.60 (12.06)	16.90 (16.77)	9.35 (11.18)	19.68 (15.96)	18.10 (14.05)	23.28 (22.46)	13.04 (16.93)	0.114

Mean (SD). QOL score comparisons among Age or Gender were performed using unpaired two tailed t-test. Comparisons between chemotherapy regimens and QOL scores were performed using Kruskal-Wallis test. *P<0.05, **P<0.01. P value in the table showed difference between QOL scores and regimens.

**Table 4:**
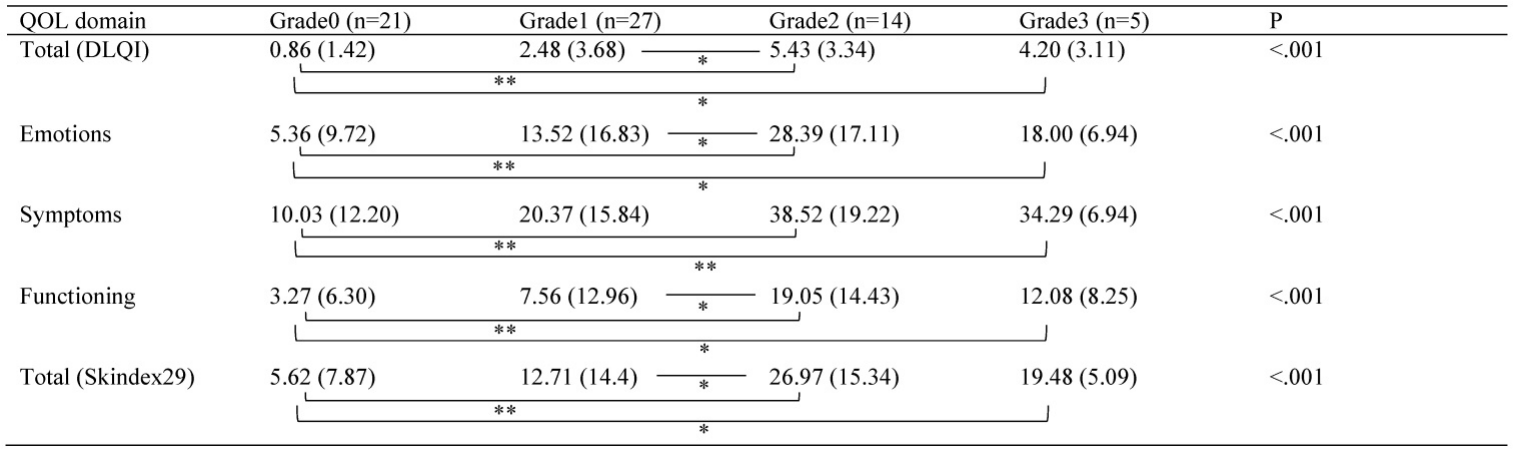
QOL Scores by Worst Skin Toxicity Grade

Mean (SD). Comparisons between QOL scores and worst skin toxicity grades were performed using Kruskal-Wallis test. Mann-Whitney Bonferroni correction was performed for comparisons among significantly different QOL domains. *P<0.05/6, **P<0.01/6

**Table 5:** Associations between QOL Scores and Skin Toxicities

	Rash	Xerosis	Paronychia	Pigmentation	Hand foot syndrome
	OR (95%Cl)	OR (95%Cl)	OR (95%Cl)	OR (95%Cl)	OR (95%Cl)
Demographic adjusted models					
Total (DLQI)	1.738 (0.681-4.434)	3.288 (1.379-7.840) **	2.161 (1.071-4.359) *	5.051 (1.193-21.386) *	2.718 (1.375-5.372) **
Emotions	2.859 (1.000-8.174)	1.928 (0.893-4.161)	1.909 (0.972-3.749)	3.040 (0.790-11.700)	2.598 (1.328-5.083) **
Symptoms	2.062 (0.785-5.417)	3.174 (1.344-7.496) **	2.298 (1.110-4.757) *	3.370 (0.869-13.061)	5.406 (2.154-13.571) **
Functioning	1.920 (0.731-5.044)	1.757 (0.815-3.791)	3.100 (1.335-7.201) **	2.311 (0.623-8.578)	2.836 (1.399-5.747) **
Total (Skindex29)	1.946 (0.706-5.364)	2.067 (0.910-4.694)	3.288 (1.310-8.251) *	2.129 (0.546-8.307)	5.354 (1.998-14.345) **
Fully adjusted models					
Total (DLQI)	1.091 (0.344-3.459)	1.749 (0.622-4.914)	1.509 (0.641-3.552)	3.077 (0.616-15.366)	2.023 (0.956-4.283)
Emotions	2.709 (0.808-9.082)	0.835 (0.295-2.365)	1.110 (0.456-2.703)	2.420 (0.499-11.734)	2.509 (1.127-5.586) *
Symptoms	1.443 (0.376-5.537)	1.512 (0.466-4.901)	1.514 (0.550-4.166)	1.794 (0.338-9.530)	4.511 (1.721-11.825) **
Functioning	1.312 (0.388-4.433)	0.705 (0.225-2.206)	2.543 (0.964-6.711)	1.958 (0.408-9.394)	2.545 (1.115-5.812) *
Total (Skindex29)	1.313 (0.328-5.252)	0.822 (0.228-2.971)	2.732 (0.922-8.090)	1.187 (0.206-6.849)	5.203 (1.698-15.943) **

Logistic regression was performed. OR, odds ratio; CI, confidence interval. *p<0.05, **p<0.01. The demographic adjusted models were adjusted for age, gender, and regimens. The fully adjusted models were additionally adjusted for rash, xerosis, paronychia, pigmentation, and hand foot syndrome.

## References

[1] McLellan B, Ciardiello F, Lacouture ME, Segaert S, Van Cutsem E (2015). Regorafenib-associated hand-foot skin reaction: practical advice on diagnosis, prevention, and management. Ann Oncol.

[2] Gunnarsson O, Pfanzelter NR, Cohen RB, Keefe SM (2015). Evaluating the safety and efficacy of axitinib in the treatment of advanced renal cell carcinoma. Cancer Manag Res.

[3] Twelves C, Wong A, Nowacki MP, Abt M, Burris H 3rd, Carrato A, Cassidy J, Cervantes A, Fagerberg J, Georgoulias V, Husseini F, Jodrell D, Koralewski P, Kröning H, Maroun J, Marschner N, McKendrick J, Pawlicki M, Rosso R, Schüller J, Seitz JF, Stabuc B, Tujakowski J, Van Hazel G, Zaluski J, Scheithauer W (2005). Capecitabine as adjuvant treatment for stage III colon cancer. N Engl J Med.

[4] Hirsh V (2011). Managing treatment-related adverse events associated with egfr tyrosine kinase inhibitors in advanced non-small-cell lung cancer. Curr Oncol.

[5] Lacouture ME, Anadkat MJ, Bensadoun RJ, Bryce J, Chan A, Epstein JB, Eaby-Sandy B, Murphy BA (2011). Clinical practice guidelines for the prevention and treatment of EGFR inhibitor-associated dermatologic toxicities. Support Care Cancer.

[6] Dai J, Belum VR, Wu S, Sibaud V, Lacouture ME (2017). Pigmentary changes in patients treated with targeted anticancer agents: A systematic review and meta-analysis. J Am Acad Dermatol.

[7] Fernandez-Peñas P, Jones-Caballero M, Espallardo O, García-Díez A (2012). Comparison of Skindex-29, Dermatology Life Quality Index, Psoriasis Disability Index and Medical Outcome Study Short Form 36 in patients with mild to severe psoriasis. Br J Dermatol.

[8] Shikiar R, Willian MK, Okun MM, Thompson CS, Revicki DA (2006). The validity and responsiveness of three quality of life measures in the assessment of psoriasis patients: results of a phase II study. Health Qual Life Outcomes.

[9] Fukuhara S (2004). Measuring HRQOL of patients with skin disease. Manual of DLQI and Skindex29 Japanese version.

[10] Pinto C, Di Fabio F, Rosati G, Lolli IR, Ruggeri EM, Ciuffreda L, Ferrari D, Lo Re G, Rosti G, Tralongo P, Ferrara R, Alabiso O, Chiara S, Ianniello GP, Frassoldati A, Bilancia D, Campanella GA, Signorelli C, Racca P, Benincasa E, Stroppolo ME, Di Costanzo F (2016). Observational study on quality of life, safety, and effectiveness of first-line cetuximab plus chemotherapy in KRAS wild-type metastatic colorectal cancer patients: the ObservEr Study. Cancer Med.

[11] Lacouture ME, Mitchell EP, Piperdi B, Pillai MV, Shearer H, Iannotti N, Xu F, Yassine M (2010). Skin toxicity evaluation protocol with panitumumab (STEPP), a phase II, open-label, randomized trial evaluating the impact of a pre-Emptive Skin treatment regimen on skin toxicities and quality of life in patients with metastatic colorectal cancer. J Clin Oncol.

[12] Barbu MA, Niţipir C, Voiosu T, Giurcăneanu C (2018). Impact of dermatologic adverse reactions on QOL in oncologic patients: results from a single-center prospective study. Rom J Intern Med.

[13] Joshi SS, Ortiz S, Witherspoon JN, Rademaker A, West DP, Anderson R, Rosenbaum SE, Lacouture ME (2010). Effects of epidermal growth factor receptor inhibitor-induced dermatologic toxicities on quality of life. Cancer.

[14] Thaler J, Karthaus M, Mineur L, Greil R, Letocha H, Hofheinz R, Fernebro E, Gamelin E, Baños A, Köhne CH (2012). Skin toxicity and quality of life in patients with metastatic colorectal cancer during first-line panitumumab plus FOLFIRI treatment in a single-arm phase II study. BMC Cancer.

[15] De Tursi M, Zilli M, Carella C, Auriemma M, Lisco MN, Di Nicola M, Di Martino G, Natoli C, Amerio P (2017). Skin toxicity evaluation in patients treated with cetuximab for metastatic colorectal cancer: a new tool for more accurate comprehension of quality of life impacts. Onco Targets Ther.

[16] Ra HS, Shin SJ, Kim JH, Lim H, Cho BC, Roh MR (2013). The impact of dermatological toxicities of anti-cancer therapy on the dermatological quality of life of cancer patients. J Eur Acad Dermatol Venereol.

[17] Koukakis R, Gatta F, Hechmati G, Siena S (2016). Skin toxicity and quality of life during treatment with panitumumab for RAS wild-type metastatic colorectal carcinoma: results from three randomised clinical trials. Qual Life Res.

[18] Iwamoto S, Ooki A, Morita S, Hara H, Tanioka H, Satake H, Kataoka M, Kotaka M, Kagawa Y, Nakamura M, Shingai T, Ishikawa M, Miyake Y, Sudo T, Hashiguchi Y, Yabuno T, Sakamoto J, Tsuji A, Ando M, Yamaguchi K (2018). A prospective Phase II study to examine the relationship between quality of life and adverse events of first-line chemotherapy plus cetuximab in patients with KRAS wild-type unresectable metastatic colorectal cancer: QUACK trial. Cancer Med.

[19] Rosen AC, Case EC, Dusza SW, Balagula Y, Gordon J, West DP, Lacouture ME (2013). Impact of dermatologic adverse events on quality of life in 283 cancer patients: a questionnaire study in a dermatology referral clinic. Am J Clin Dermatol.

[20] Kozuki T (2016). Skin problems and EGFR-tyrosine kinase inhibitor. Jpn J Clin Oncol.

[21] Lüftner D, Dell'Acqua V, Selle F, Khalil A, Leonardi MC, De La Torre Tomás A, Shenouda G, Romero Fernandez J, Orecchia R, Moyal D, Seité S (2018). Evaluation of supportive and barrier-protective skin care products in the daily prevention and treatment of cutaneous toxicity during systemic chemotherapy. Onco Targets Ther.

[22] Cirillo M, Venturini M, Ciccarelli L, Coati F, Bortolami O, Verlato G (2009). Clinician versus nurse symptom reporting using the National Cancer Institute-Common Terminology Criteria for Adverse Events during chemotherapy: results of a comparison based on patient's self-reported questionnaire. Ann Oncol.

